# A Time Two-Mesh Compact Difference Method for the One-Dimensional Nonlinear Schrödinger Equation

**DOI:** 10.3390/e24060806

**Published:** 2022-06-09

**Authors:** Siriguleng He, Yang Liu, Hong Li

**Affiliations:** 1 School of Mathematics and Big Data, Hohhot Minzu College, Hohhot 010051, China; hsr@imu.edu.cn; 2 School of Mathematical Sciences, Inner Mongolia University, Hohhot 010021, China; smslh@imu.edu.cn

**Keywords:** high-order compact difference scheme, time two-mesh algorithm, error estimate, conservation law, soliton

## Abstract

The nonlinear Schrödinger equation is an important model equation in the study of quantum states of physical systems. To improve the computing efficiency, a fast algorithm based on the time two-mesh high-order compact difference scheme for solving the nonlinear Schrödinger equation is studied. The fourth-order compact difference scheme is used to approximate the spatial derivatives and the time two-mesh method is designed for efficiently solving the resulting nonlinear system. Comparing to the existing time two-mesh algorithm, the novelty of the new algorithm is that the fine mesh solution, which becomes available, is also used as the initial guess of the linear system, which can improve the calculation accuracy of fine mesh solutions. Compared to the two-grid finite element methods (or finite difference methods) for nonlinear Schrödinger equations, the numerical calculation of this method is relatively simple, and its two-mesh algorithm is implemented in the temporal direction. Taking advantage of the discrete energy, the result with $O(\tau_{C}^{4}+\tau_{F}^{2}+h^{4})$ in the discrete $L_{2}$-norm is obtained. Here, $\tau_{C}$ and $\tau_{F}$ are the temporal parameters on the coarse and fine mesh, respectively, and *h* is the space step size. Finally, some numerical experiments are conducted to demonstrate its efficiency and accuracy. The numerical results show that the new algorithm gives highly accurate results and preserves conservation laws of charge and energy. Furthermore, by comparing with the standard nonlinear implicit compact difference scheme, it can reduce the CPU time without loss of accuracy.

## 1. Introduction

In this paper, we consider the one-dimensional cubic nonlinear Schrödinger (NLS) equation

(1) $$i\frac{\partial u}{\partial t}+\eta \frac{\partial^{2}u}{\partial x^{2}}+q{\left|u\right|}^{2}u=0,\;x\in R,\;t\geq 0,$$

where $i=\sqrt{-1}$ is the complex unit, the subscripts *x* and *t* denote the spatial and temporal variable, respectively; η and *q* are positive real constants; u=u(x,t) is an unknown complex-valued wave function. The initial condition

u(x,0)=φ(x),x∈R,

is a prescribed smooth complex function, which decreases exponentially as |x|→0. We assume that the solution to NLS Equation (1) has compact support on a bounded interval [a,b] during the time period [0,T]. So, artificial boundary conditions

u(a,t)=u(b,t)=0,t∈(0,T],

are taken here.

The NLS equation is one of the most important equations of mathematical physics and it has been widely used to model various nonlinear physical phenomena, such as underwater acoustics, plasma physics, bimolecular dynamics, and nonlinear optics. NLS Equation (1) is a generic model for the slowly varying envelop of a wave train in conservative, dispersive, mildly nonlinear wave phenomena. It is also obtained as the subsonic limit of the Zakharov model for Langmuir waves in plasma physics, and known as the Gross–Pitaevskii equation (GPE) in modeling the hydrodynamics of the Bose–Einstein condensate [1]. The real constant parameter *q* in Equation (1) (focusing for q>0, and defocusing for q<0) describes the strength of the local interactions between particles. The complex function u(x,t) describes the envelope of a physical solution, and, in optics, its squared modulus represents a measurable quantity, viz. intensity. Localizing along the *t* axis, NLS Equation (1) has soliton solutions, which can exist on a zero background (completely localized) or on a plane wave background [2]. Localizing along the *x* axis, it has the “Akhmediev breathers” [2,3]. The interrelation between these solutions is schematically represented in [2]. The extended family of NLS equation contains the Hirota equation (HE) and other higher-order members of the NLS hierarchy of equations [4,5]. In [4,5], the numerical simulations with high accuracy along the transversal axis were used to calculate solitons and breathers of the Hirota equation, which is an extension of the NLS equation.

Due to the presence of nonlinearity and the complex nature of the NLS equation, it is still a challenge for researchers to determine the most suitable method. Many analytical and numerical studies have been carried out to overcome this difficulty. Along the analytical front, one can refer to [3,6] and the references therein. Along the numerical front, different efficient and accurate numerical methods including finite difference (FD) methods [7,8,9,10,11,12,13,14,15,16,17], finite element (FE) methods [18,19,20,21,22], spectral method [23], discontinuous Galerkin method [24], virtual element method [25], and so on. Furthermore, there have also been some other efficient algorithms, such as the multigrid methods [26,27,28] and two-grid methods [29,30,31,32,33,34,35,36,37], where the idea is presented firstly by Xu in [38]. In [29,30,31,32,33], some two-grid (mixed) finite element schemes were proposed for solving the NLS equation, respectively, and the error estimates are discussed. In [34,35], Zhang et al. and Chen et al. constructed two-grid finite volume (element) methods and performed the corresponding convergence analysis, respectively. In [36], Wang et al. discussed the global $H_{1}$-norm super-convergence result of the two-grid FE method for the NLS equation. Ignat et al. [37] constructed a space two-grid FD scheme for NLS equations, where the equations on the fine grid are linearized, but not decoupled.

It is worth mentioning that compared with the two-grid FEMs [29,30,31,32,33,34,35,36], the two-grid FDMs [37,39,40,41] are relatively simple from the point of view of numerical calculation. This means that the time two-mesh (TT-M) method combined with FD can also solve the NLS equation with better computational efficiency. The TT-M algorithm is proposed firstly by Liu et al. [42], and combined with the FE method to solve some other fractional models [43,44,45]. Recently, based on the idea proposed in [42], Qiu and Xu et al. [46,47] developed and analyzed a TT-M algorithm based on FD methods for nonlinear fractional partial differential equations (FPDEs). Niu et al. [48] and Chai et al. [49] used the TT-M technique to propose a fast high-order compact difference scheme for the nonlinear distributed-order fractional Sobolev model appearing in porous media and nonlinear space fractional Gray–Scott model, respectively; however, from the current literature, we find that there is no report about the TT-M based on the compact difference (CD) method for solving the NLS equation.

The aim of this paper is the development of a time two-mesh high-order compact difference (TT-MCD) method to obtain the solution of the NLS Equation (1). We show that the TT-MCD method is suitable for the treatment of the NLS equation. In addition, for the study of the two-grid FD method, our article is different from Refs. [37,39,40,41], where a two-grid algorithm for the spatial direction is combined with the finite difference method; however, our article uses a two-mesh algorithm for the temporal direction. Furthermore, compared to the TT-M algorithms [42,43,44,46,47], our method made a modification, analogous to the Gauss–Seidel method for linear systems, of the initial guess in the linearization process, where the available fine grid solution is used as well. Such modification can improve the calculation accuracy of fine grid solutions. In addition, through numerical schemes and experiments, it can find that our method has the advantages of the simple numerical calculation. In addition, compared to the standard nonlinear implicit (SNI) CD scheme, it can reduce the CPU time without loss of accuracy.

The new TT-MCD algorithm includes three steps: firstly, a nonlinear implicit CD scheme, which will be solved by an iterative method on the time coarse mesh, is established; secondly, utilizing the numerical data obtained from the first step, the Lagrange’s linear interpolation formula is employed to obtain rough solutions on the time fine mesh; finally, one Newton iteration is applied on the time fine mesh to linearize the nonlinear CD scheme using the mean value of the rough solution and available fine mesh solution as the initial guess. We then solve the linear system to obtain the finial numerical solutions. The main contributions or contents of this article are as follows:

- A fast numerical algorithm, which is formulated by combining the fourth-order CD method with the TT-M method, is proposed and developed to solve the NLS equation.
- A modification, analogous to the Gauss–Seidel method for linear systems, of the initial guess in the linearization process is taken to improve the calculation accuracy of fine grid solutions.
- By using the discrete energy, the detailed proof of the convergence result with $O(\tau_{C}^{4}+\tau_{F}^{2}+h^{4})$ in the discrete $L_{2}$-norm is given.
- Numerical experiments on some model problems, including single soliton, interaction of two solitons and birth of standing soliton, are conducted to demonstrate efficiency and accuracy of the TT-MCD algorithm.
- It is easy to see from the numerical results that the proposed TT-MCD algorithm not only gives highly accurate results and preserves conservation laws of charge and energy, but also can save the CPU time.

The remainder of this paper is organized as follows. In Section 2, notations and some lemmas are given. Section 3 devotes to the establishment of the time two-mesh compact difference scheme. The convergence of the TT-MCD scheme is analyzed in Section 4. In Section 5, two numerical examples are given to verify the feasibility and effectiveness. The article ends with a brief conclusions section. Throughout this paper, the symbol *M* is used to denote a generic positive constant.

## 2. Notations and Some Lemmas

First, for the temporal approximation on the fine mesh $T_{F}$, we define $\tau_{F}=\frac{T}{N},$$t_{n}=n\tau_{F}\;\left(n=0,1,\cdots ,N\right)$. Similarly, for the coarse mesh $T_{C}$, denote $\tau_{C}=\frac{T}{N}$ where $N=\frac{N}{s},\;\left(2\leq s\in Z^{+}\right),\;t_{ks}=k\tau_{C}\;\left(k=0,1,\cdots ,N\right)$. For the spatial approximation, let $h=\frac{b-a}{J}$ for positive integer *J*, $x_{j}=a+jh,\;j=0,1,\cdots ,J$. Let $u_{j}^{n}=u\left(x_{j},t_{n}\right)$ and $I_{h}=\left\{x_{0},x_{1},\cdots ,x_{J}\right\}$ denote the set of nodes of the interval [a,b]. We use the following notations for simplicity:

$$\begin{array}{cc} \; & {\left(u_{j}\right)}_{x}^{n}=\frac{u_{j+1}^{n}-u_{j}^{n}}{h},\;{\left(u_{j}\right)}_{\bar{x}}^{n}=\frac{u_{j}^{n}-u_{j-1}^{n}}{h},\;{\left(u_{j}\right)}_{t}^{n}=\frac{u_{j}^{n+1}-u_{j}^{n}}{\tau },\\ \; & {\left(u_{j}\right)}_{x\bar{x}}^{n}=\frac{u_{j+1}^{n}-2u_{j}^{n}+u_{j-1}^{n}}{h^{2}},\;u_{j}^{n+\frac{1}{2}}=\frac{1}{2}\left(u_{j}^{n+1}+u_{j}^{n}\right), \end{array}$$

where τ denotes the time step length $\tau_{C}$ or $\tau_{F}$. Let $H_{h,0}$ denote the set of mesh functions *u* defined on $I_{h}$ with boundary conditions $u_{0}=u_{j}=0$. We define the discrete inner products and norms via

$$\begin{array}{cc} \; & \left(u,w\right)=\sum_{j=1}^{J-1}u_{j}{\bar{w}}_{j}h,\;{\left(u_{x},w_{x}\right)}_{l}=\sum_{j=0}^{J-1}{\left(u_{j}\right)}_{x}{\left({\bar{w}}_{j}\right)}_{x}h\;\forall u,w\in H_{h,0},\\ \; & {∥u∥}_{L_{2}}=\sqrt{\left(u,u\right)}{,\;∥u∥}_{L_{\infty }}=\max_{1\leq j\leq J-1}|u_{j}\left|,\;∥\right|u_{x}{|∥}_{L_{2}}=\sqrt{{\left(u_{x},u_{x}\right)}_{l}}. \end{array}$$

And for any complex-valued function u=v+iw, let

$${∥u∥}_{L_{2}}={\left({∥v∥}_{L_{2}}^{2}+{∥w∥}_{L_{2}}^{2}\right)}^{1/2},\;\;∥|u_{x}{|∥}_{L_{2}}={\left(∥|v_{x}{|∥}_{L_{2}}^{2}+∥|w_{x}{|∥}_{L_{2}}^{2}\right)}^{1/2}.$$

Next, we give some auxiliary lemmas, which will be used later.

**Lemma** **1** (See [7]). *For any grid functions $u,w\in H_{h,0}$, we have*

$$\left(a\right)\;\left(u_{x\bar{x}},w\right)=-{\left(u_{x},w_{x}\right)}_{l},\;\;\left(b\right)\;∥|u_{x}{|∥}_{L_{2}}^{2}\leq \frac{4}{h^{2}}{∥u∥}_{L_{2}}^{2},\;\;\left(c\right){\;∥u∥}_{L_{\infty }}\leq \frac{\sqrt{b-a}}{2}∥|u_{x}{|∥}_{L_{2}}.$$

**Lemma** **2** (See [7]). *Assume that a sequence of nonnegative real numbers ${\left\{a_{j}\right\}}_{j=0}^{\infty }$ satisfying*

$$a_{n+1}\leq \alpha +\beta \sum_{j=0}^{n}a_{j}\tau ,\;n\geq 0,$$

*then there has the inequality $a_{n+1}\leq \left(\alpha +\tau \beta a_{0}\right)e^{\beta (n+1)\tau }$, where α≥0,β and τ are positive constants.*

## 3. The Time Two-Mesh Compact Difference Scheme

In order to construct the compact difference scheme [7], we first split the NLS Equation (1) into a system

$$\left\{\begin{array}{l} -i\frac{\partial u}{\partial t}-q{\left|u\right|}^{2}u=\nu ,\\ \eta \frac{\partial^{2}u}{\partial x^{2}}=\nu . \end{array}\right.$$

Using Taylor expansion, we obtain

(2) $$-i{\left(u_{j}\right)}_{t}^{n}-{q(|u|}^{2}{)}_{j}^{n+\frac{1}{2}}u_{j}^{n+\frac{1}{2}}=\nu_{j}^{n+\frac{1}{2}}+O\left(\tau^{2}\right),$$

and

(3) $$\begin{array}{cc} \nu_{j}^{n+\frac{1}{2}} & =\eta {\left(\frac{\partial^{2}u}{\partial x^{2}}\right)}_{j}^{n+\frac{1}{2}}=\eta {\left(u_{j}\right)}_{x\bar{x}}^{n+\frac{1}{2}}-\eta \frac{h^{2}}{12}{\left(\frac{\partial^{4}u}{\partial x^{4}}\right)}_{j}^{n+\frac{1}{2}}+O\left(h^{4}\right)\\ \; & =\eta {\left(u_{j}\right)}_{x\bar{x}}^{n+\frac{1}{2}}-\frac{h^{2}}{12}{\left(\frac{\partial^{2}\nu }{\partial x^{2}}\right)}_{j}^{n+\frac{1}{2}}+O\left(h^{4}\right)\\ \; & =\eta {\left(u_{j}\right)}_{x\bar{x}}^{n+\frac{1}{2}}-\frac{h^{2}}{12}{\left(\nu_{j}\right)}_{x\bar{x}}^{n+\frac{1}{2}}+O\left(h^{4}\right). \end{array}$$

From Equations (2) and (3), we obtain

(4) $$i{\left(u_{j}\right)}_{t}^{n}+{q(|u|}^{2}{)}_{j}^{n+\frac{1}{2}}u_{j}^{n+\frac{1}{2}}+\eta {\left(u_{j}\right)}_{x\bar{x}}^{n+\frac{1}{2}}-\frac{1}{12}\left(\nu_{j+1}^{n+\frac{1}{2}}-2\nu_{j}^{n+\frac{1}{2}}+\nu_{j-1}^{n+\frac{1}{2}}\right)=O\left(\tau^{2}+h^{4}\right).$$

Then substituting Equations (2) into (4), we have

(5) $$\begin{array}{cc} \; & \frac{i}{12}\left[{\left(u_{j-1}\right)}_{t}^{n}+10{\left(u_{j}\right)}_{t}^{n}+{\left(u_{j+1}\right)}_{t}^{n}\right]+\eta {\left(u_{j}\right)}_{x\bar{x}}^{n+\frac{1}{2}}\\ \; & \;+\frac{q}{12}\left[{\left(|u\right|}^{2}{)}_{j-1}^{n+\frac{1}{2}}u_{j-1}^{n+\frac{1}{2}}+{10(|u|}^{2}{)}_{j}^{n+\frac{1}{2}}u_{j}^{n+\frac{1}{2}}+{\left(|u\right|}^{2}{)}_{j+1}^{n+\frac{1}{2}}\right]\\ \; & \;=O(\tau^{2}+h^{4}), \end{array}$$

Next, based on Equation (5), a time two-mesh CD scheme for problem Equation (1) is constructed as follows.

Step 1: Letting $U_{C,j}^{ks}=V_{C,j}^{ks}+iW_{C,j}^{ks}$, we find $\left\{V_{C,j}^{ks+1},W_{C,j}^{ks+1}\right\}\in H_{h,0}\times H_{h,0}$ on the coarse mesh, such that

(6) $$\begin{array}{cc} \; & -\frac{1}{12}\left[{\left(W_{C,j+1}\right)}_{t}^{ks}+10{\left(W_{C,j}\right)}_{t}^{ks}+{\left(W_{C,j-1}\right)}_{t}^{ks}\right]+\eta {\left(V_{C,j}\right)}_{x\bar{x}}^{ks+\frac{1}{2}}\\ \; & \;\;+\frac{q}{12}\left[{\hat{F}}_{j-1}+10{\hat{F}}_{j}+{\hat{F}}_{j+1}\right]=0,\;1\leq j\leq J-1, \end{array}$$

(7) $$\begin{array}{cc} \; & \frac{1}{12}\left[{\left(V_{C,j-1}\right)}_{t}^{ks}+10{\left(V_{C,j}\right)}_{t}^{ks}+{\left(V_{C,j+1}\right)}_{t}^{ks}\right]+\eta {\left(W_{C,j}\right)}_{x\bar{x}}^{ks+\frac{1}{2}}\\ \; & \;\;+\frac{q}{12}\left[{\tilde{F}}_{j-1}+10{\tilde{F}}_{j}+{\tilde{F}}_{j+1}\right]=0,\;1\leq j\leq J-1, \end{array}$$

(8) $$\begin{array}{cc} \; & U_{C,j}^{0}=\varphi \left(x_{j}\right),\;\;\;1\leq j\leq J-1,\\ \; & V_{C,0}^{ks}=V_{C,J}^{ks}=0,\;\;W_{C,0}^{ks}=W_{C,J}^{ks}=0,\;\;\;1\leq k\leq N, \end{array}$$

where

$$\begin{array}{c} {\hat{F}}_{j}=\left(\right|U_{C,j}{|}^{2}{)}^{ks+\frac{1}{2}}V_{C,j}^{ks+\frac{1}{2}},\;\;{\tilde{F}}_{j}=\left(\right|U_{C,j}{{|}^{2})}^{ks+\frac{1}{2}}W_{C,j}^{ks+\frac{1}{2}}. \end{array}$$

Step 2: Based on the solutions $U_{C,j}^{ks}$ obtained from Step 1, we use the Lagrange’s linear interpolation formula to compute $U_{C,j}^{n}\;\left(n=1,2,\cdots ,N\right)$, that is, at time levels $t_{ks-l}\;\left(l=0,1,\cdots ,\;k,\mathrm{and}\;k=1,2,\cdots ,N,\;ks-l=n\right)$, we have

(9) $$\begin{array}{cc} U_{C}^{ks-l} & =\frac{t_{ks-l}-t_{ks}}{t_{(k-1)s}-t_{ks}}U_{C}^{(k-1)s}+\frac{t_{ks-l}-t_{(k-1)s}}{t_{ks}-t_{(k-1)s}}U_{C}^{ks}\\ \; & =\frac{l}{s}U_{C}^{(k-1)s}+\left(1-\frac{l}{s}\right)U_{C}^{ks}. \end{array}$$

Step 3: Taking the mean value of the solution $U_{C}^{n+1}$ obtained from Step 2 and the former time level fine mesh solution $U_{F}^{n}$ as the initial value, we construct a linear system on the time fine mesh as follows to solve the solutions $\left\{V_{F,j}^{n+1},W_{F,j}^{n+1}\right\}\in H_{h,0}\times H_{h,0}$ such that

(10) $$\begin{array}{cc} \; & \frac{1}{12}\left[{\left(W_{F,j+1}\right)}_{t}^{n}+10{\left(W_{F,j}\right)}_{t}^{n}+{\left(W_{F,j-1}\right)}_{t}^{n}\right]-\eta {\left(V_{F,j}\right)}_{x\bar{x}}^{n+\frac{1}{2}}-\frac{q}{12}\left[{\hat{\Theta }}_{j-1}+10{\hat{\Theta }}_{j}+{\hat{\Theta }}_{j+1}\right]=0, \end{array}$$

(11) $$\begin{array}{cc} \; & \frac{1}{12}[{\left(V_{F,j-1}\right)}_{t}^{n}+10{\left(V_{F,j}\right)}_{t}^{n}+{\left(V_{F,j+1}\right)}_{t}^{n}]+\eta {\left(W_{F,j}\right)}_{x\bar{x}}^{n+\frac{1}{2}}+\frac{q}{12}\left[{\tilde{\Theta }}_{j-1}+10{\tilde{\Theta }}_{j}+{\tilde{\Theta }}_{j+1}\right]=0, \end{array}$$

(12) $$\begin{array}{cc} \; & U_{F,j}^{0}=\varphi \left(x_{j}\right),\;V_{F,0}^{n}=V_{F,J}^{n}=0,\;W_{F,0}^{n}=W_{F,J}^{n}=0\;1\leq j\leq J-1,0\leq n\leq N, \end{array}$$

where

$$\begin{array}{cc} {\hat{\Theta }}_{j} & =\frac{1}{8}\left[{\left(V_{C,j}^{n+1}+V_{F,j}^{n}\right)}^{2}+(W_{C,j}^{n+1}+{\left(W_{F,j}^{n}\right)}^{2}\right]\left(V_{C,j}^{n+1}+V_{F,j}^{n}\right)\\ \; & +\frac{1}{8}\left[3{\left(V_{C,j}^{n+1}+V_{F,j}^{n}\right)}^{2}+{\left(W_{C,j}^{n+1}+W_{F,j}^{n}\right)}^{2}\right]\left(V_{F,j}^{n+1}-V_{C,j}^{n+1}\right)\\ \; & +\frac{1}{4}\left[\left(V_{C,j}^{n+1}+V_{F,j}^{n}\right)\left(W_{C,j}^{n+1}+W_{F,j}^{n}\right)\right]\left(W_{F,j}^{n+1}-W_{C,j}^{n+1}\right), \end{array}$$

$$\begin{array}{cc} {\tilde{\Theta }}_{j}\; & =\frac{1}{8}\left[{\left(V_{C,j}^{n+1}+V_{F,j}^{n}\right)}^{2}+(W_{C,j}^{n+1}+{\left(W_{F,j}^{n}\right)}^{2}\right]\left(W_{C,j}^{n+1}+W_{F,j}^{n}\right)\\ \; & +\frac{1}{8}\left[3{\left(W_{C,j}^{n+1}+W_{F,j}^{n}\right)}^{2}+{\left(V_{C,j}^{n+1}+V_{F,j}^{n}\right)}^{2}\right]\left(W_{F,j}^{n+1}-W_{C,j}^{n+1}\right)\\ \; & +\frac{1}{4}\left[\left(V_{C,j}^{n+1}+V_{F,j}^{n}\right)\left(W_{C,j}^{n+1}+W_{F,j}^{n}\right)\right]\left(V_{F,j}^{n+1}-V_{C,j}^{n+1}\right). \end{array}$$

**Remark** **1.** *A modification, analogous to the Gauss–Seidel method for linear systems, of our algorithm is that the fine mesh solution $U_{F}^{n}$ is also used in calculation of the fine mesh solution $U_{F}^{n+1}$, by which one can improve the calculation accuracy of fine mesh solutions.*

## 4. The Convergence Analysis of the TT-MCD Scheme

In this section, we first consider error analysis of the nonlinear system on the time coarse mesh. Denote $e_{C,j}^{ks}=v_{j}^{ks}-V_{C,j}^{ks},\;E_{C,j}^{ks}=w_{j}^{ks}-W_{C,j}^{ks},R_{C,j}^{ks}={\hat{R}}_{C,j}^{ks}+i{\tilde{R}}_{C,j}^{ks},\;1\leq j\leq J-1,$0≤k≤N. From Equations (5)–(7), we obtain

(13) $$\begin{array}{cc} \; & -\frac{h^{2}}{12}{\left(E_{C,j}\right)}_{t,x\bar{x}}^{ks}-{\left(E_{C,j}\right)}_{t}^{ks}+\eta {\left(e_{C,j}\right)}_{x\bar{x}}^{ks+\frac{1}{2}}+\frac{qh^{2}}{12}[g{\left(v_{j}^{ks+\frac{1}{2}},w_{j}^{ks+\frac{1}{2}}\right)}_{x\bar{x}}\\ \; & \;-g{\left(V_{C,j}^{ks+\frac{1}{2}},W_{C,j}^{ks+\frac{1}{2}}\right)}_{x\bar{x}}]+q\left[g\left(v_{j}^{ks+\frac{1}{2}},w_{j}^{ks+\frac{1}{2}}\right)-g\left(V_{C,j}^{ks+\frac{1}{2}},W_{C,j}^{ks+\frac{1}{2}}\right)\right]={\hat{R}}_{C,j}^{ks}, \end{array}$$

(14) $$\begin{array}{cc} \; & \frac{h^{2}}{12}{\left(e_{C,j}\right)}_{t,x\bar{x}}^{ks}+{\left(e_{C,j}\right)}_{t}^{ks}+\eta {\left(E_{C,j}\right)}_{x\bar{x}}^{ks+\frac{1}{2}}+\frac{qh^{2}}{12}[g\left(w_{j}^{ks+\frac{1}{2}},v_{j}^{ks+\frac{1}{2}}\right)\\ \; & \;-g{\left(W_{C,j}^{ks+\frac{1}{2}},V_{C,j}^{ks+\frac{1}{2}}\right)}_{x\bar{x}}]+q\left[g\left(w_{j}^{ks+\frac{1}{2}},v_{j}^{ks+\frac{1}{2}}\right)-g\left(W_{C,j}^{ks+\frac{1}{2}},V_{C,j}^{ks+\frac{1}{2}}\right)\right]={\tilde{R}}_{C,j}^{ks}, \end{array}$$

where ${\hat{R}}_{C}^{ks}=O\left(\tau_{C}^{2}+h^{4}\right),\;{\tilde{R}}_{C}^{ks}=O\left(\tau_{C}^{2}+h^{4}\right)$, and the function $g\left(x,y\right)=\left(x^{2}+y^{2}\right)x$ satisfies $\max_{\left(x,y\right)\in R^{2}}|g_{x}\left(x,y\right)|+|g_{y}\left(x,y\right)|\leq L.$

For simplification, we further denote ϕ=g(v,w)−g(V,W) and ψ=g(w,v)−g(W,V). Then Equations (13) and (14) can be written as

(15) $$-\frac{h^{2}}{12}{\left(E_{C,j}\right)}_{t,x\bar{x}}^{ks}-{\left(E_{C,j}\right)}_{t}^{ks}+\eta {\left(e_{C,j}\right)}_{x\bar{x}}^{ks}+\frac{qh^{2}}{12}{\left(\phi_{C,j}\right)}_{x\bar{x}}^{ks+\frac{1}{2}}+q\phi_{C,j}^{ks+\frac{1}{2}}={\hat{R}}_{C,j}^{ks},$$

(16) $$\frac{h^{2}}{12}{\left(e_{C,j}\right)}_{t,x\bar{x}}^{ks}+{\left(e_{C,j}\right)}_{t}^{ks}+\eta {\left(E_{C,j}\right)}_{x\bar{x}}^{ks+\frac{1}{2}}+\frac{qh^{2}}{12}{\left(\psi_{C,j}\right)}_{x\bar{x}}^{ks+\frac{1}{2}}+q\psi_{C,j}^{ks+\frac{1}{2}}={\tilde{R}}_{C,j}^{ks}.$$

Based on the above set of error equations, we obtain the following error estimation of the coarse mesh solution.

**Theorem** **1.** *Suppose that the exact solution $u^{n}=v^{n}+iw^{n}$ to the initial boundary value problem Equation (1) is sufficiently smooth and let $U_{C}^{n}=V_{C}^{n}+iW_{C}^{n}$ be the numerical solution on the time coarse mesh. Then, there exist a positive constant M independent of $h,\tau_{C}$ such that*

$$∥u^{n}-U_{C}^{n}{∥}_{L_{2}}\leq M\left(\tau_{C}^{2}+h^{4}\right).$$

**Proof.**  (I) The proof contains two cases. First, we consider the case of n=ks,(k=1,⋯,N). For simplification, we will omit the subindex *j* and the mark *C* of coarse mesh in Equations (15) and (16). Taking the inner product (·,·) on both sides of Equation (15) with $E^{n+1}+E^{n}$, we obtain

$$\begin{array}{cc} \frac{h^{2}}{12\tau } & \left[∥|E_{x}^{n+1}{|∥}_{L_{2}}^{2}-∥|E_{x}^{n}{|∥}_{L_{2}}^{2}\right]-\frac{1}{\tau }\left[∥E^{n+1}{∥}_{L_{2}}^{2}-{∥E^{n}∥}_{L_{2}}^{2}\right]\\ \; & -2\eta \left(e_{x}^{n+\frac{1}{2}},E_{x}^{n+\frac{1}{2}}\right)-\frac{qh^{2}}{6}\left(\phi_{x}^{n+\frac{1}{2}},E_{x}^{n+\frac{1}{2}}\right)+2q\left(\phi^{n+\frac{1}{2}},E^{n+\frac{1}{2}}\right)=2\left({\hat{R}}^{n},E^{n+\frac{1}{2}}\right). \end{array}$$

Using Lemma 1 and Cauchy–Schwarz inequality, we obtain

(17) $$\begin{array}{cc} \; & ∥E^{n+1}{∥}_{L_{2}}^{2}-\frac{h^{2}}{12}∥|E_{x}^{n+1}{|∥}_{L_{2}}^{2}+2\eta \tau \left(e_{x}^{n+\frac{1}{2}},E_{x}^{n+\frac{1}{2}}\right)\\ \; & \leq ∥E^{n}{∥}_{L_{2}}^{2}-\frac{h^{2}}{12}∥|E_{x}^{n}{|∥}_{L_{2}}^{2}+\frac{4q\tau }{3}∥\phi^{n+\frac{1}{2}}{∥}_{L_{2}}^{2}+\tau \left(\frac{4q}{3}+1\right)∥E^{n+\frac{1}{2}}{∥}_{L_{2}}^{2}+\tau {\left|{\hat{R}}^{n}\right|}^{2}. \end{array}$$

Using two order Taylor expansion at a point $\left(V^{n+\frac{1}{2}},W^{n+\frac{1}{2}}\right)$ for $g(v^{n+\frac{1}{2}},w^{n+\frac{1}{2}})$, we have

(18) $$\begin{array}{cc} ∥\phi^{n+\frac{1}{2}}{∥}_{L_{2}}^{2}\; & \leq 2∥g_{x}\left(v^{*},w^{*}\right)\left(v^{n+\frac{1}{2}}-V^{n+\frac{1}{2}}\right){∥}_{L_{2}}^{2}+2{∥g_{y}\left(v^{*},w^{*}\right)\left(w^{n+\frac{1}{2}}-W^{n+\frac{1}{2}}\right)∥}_{L_{2}}^{2}\\ \; & \leq 2\;L^{2}∥v^{n+\frac{1}{2}}-V^{n+\frac{1}{2}}{∥}_{L_{2}}^{2}+2L^{2}{∥w^{n+\frac{1}{2}}-W^{n+\frac{1}{2}}∥}_{L_{2}}^{2}. \end{array}$$

Combining Equation (17) with Equation (18), we then have

(19) $$\begin{array}{cc} \; & ∥E^{n+1}{∥}_{L_{2}}^{2}-\frac{h^{2}}{12}∥|E_{x}^{n+1}{|∥}_{L_{2}}^{2}+2\tau \eta \left(e_{x}^{n+\frac{1}{2}},E_{x}^{n+\frac{1}{2}}\right)\\ \; & \leq ∥E^{n}{∥}_{L_{2}}^{2}-\frac{h^{2}}{12}∥E_{x}^{n}{∥}_{L_{2}}^{2}+\frac{8\;Mq\tau }{3}∥e^{n+\frac{1}{2}}{∥}_{L_{2}}^{2}+\tau \left(4q+1\right)M∥E^{n+\frac{1}{2}}{∥}_{L_{2}}^{2}+\tau {\left|{\hat{R}}^{n}\right|}^{2}. \end{array}$$

Similarly, taking the inner product (·,·) on both sides of Equation (16) with $e^{n+1}+e^{n}$, and then using Lemma 1, Cauchy–Schwarz inequality and Taylor expansion for function *g*, we can obtain

(20) $$\begin{array}{cc} \; & ∥e^{n+1}{∥}_{L_{2}}^{2}-\frac{h^{2}}{12}∥|e_{x}^{n+1}{|∥}_{L_{2}}^{2}-2\tau \eta \left(E_{x}^{n+\frac{1}{2}},e_{x}^{n+\frac{1}{2}}\right)\\ \; & \leq ∥e^{n}{∥}_{L_{2}}^{2}-\frac{h^{2}}{12}∥|e_{x}^{n}{|∥}_{L_{2}}^{2}+\frac{8Mq\tau }{3}∥E^{n+\frac{1}{2}}{∥}_{L_{2}}^{2}+\tau \left(4q+1\right)M∥e^{n+\frac{1}{2}}{∥}_{L_{2}}^{2}+\tau {\left|{\tilde{R}}^{n}\right|}^{2}. \end{array}$$

summing from 0 to n−1 in Equations (19) and (20), respectively, and then adding the two inequalities, we have

$$\left(\frac{2}{3}-M\tau_{C}\right)∥u^{n}-U_{C}^{n}{∥}_{L_{2}}^{2}\leq M\tau_{C}\sum_{p=0}^{n-1}∥u^{p}-U_{C}^{P}{∥}_{L_{2}}^{2}+2\tau_{C}\sum_{p=0}^{n-1}{\left|R^{p}\right|}^{2}.$$

By taking $\tau_{C}$ small enough so that $\tau_{C}<\frac{2}{3M}$ and applying Lemma 2, we obtain $∥u^{n}-U_{C}^{n}{∥}_{L_{2}}\leq M\left(\tau_{C}^{2}+h^{4}\right).$ Further, noticing that n=ks, then we have

(21) $$∥u^{ks}-U_{C}^{ks}{∥}_{L_{2}}\leq M\left(\tau_{C}^{2}+h^{4}\right).$$

(II) The second case is n=ks−l,(l=1,⋯,k−1,andk=1,2,⋯,N). Based on the Lagrange’s interpolation formula, we obtain

(22) $$\begin{array}{cc} u^{ks-l} & =\frac{t_{ks-l}-t_{ks}}{t_{(k-1)s}-t_{ks}}u^{(k-1)s}+\frac{t_{ks-l}-t_{(k-1)s}}{t_{ks}-t_{(k-1)s}}u^{ks}=\frac{l}{s}u^{(k-1)s}+\left(1-\frac{l}{s}\right)u^{ks}\\ \; & +\frac{u\prime \prime \left(\eta \right)}{2}\left(t-t_{(k-1)s}\right)\left(t-t_{ks}\right),\;\eta \in \left(t_{(k-1)s},t_{ks}\right). \end{array}$$

Subtracting Equations (9) and (22), we can have

$$\begin{array}{cc} u^{ks-l}-U_{C}^{ks-l} & =\frac{l}{s}\left(u^{(k-1)s}-U_{C}^{(k-1)s}\right)+\left(1-\frac{l}{s}\right)\left(u^{ks}-U_{C}^{ks}\right)\\ \; & +\frac{u\prime \prime \left(\eta \right)}{2}\left(t-t_{(k-1)s}\right)\left(t-t_{ks}\right). \end{array}$$

Using (21) and triangle inequality, we obtain $∥u^{ks-l}-U_{C}^{ks-l}{∥}_{L_{2}}\leq M\left(\tau_{C}^{2}+h^{4}\right).$ In addition to synthesizing the above two cases, we then obtain the result of Theorem 1. □

Next, we give the convergence result on the time fine mesh. Letting $e_{F,j}^{n}=v_{j}^{n}-V_{F,j}^{n},$$\;E_{F,j}^{n}=w_{j}^{n}-W_{F,j}^{n},\;\left(1\leq j\leq J-1,0\leq n\leq N\right)$, then from Equations (5), (10) and (11), we obtain

(23) $$\begin{array}{cc} -{\left(E_{F,j}\right)}_{t}^{n}-\frac{h^{2}}{12}{\left(E_{F,j}\right)}_{t,x\bar{x}}^{n}\; & +\eta {\left(e_{F,j}\right)}_{x\bar{x}}^{n+\frac{1}{2}}+\frac{qh^{2}}{12}{\left(g\left(v_{j}^{n+\frac{1}{2}},w_{j}^{n+\frac{1}{2}}\right)-{\hat{\Theta }}_{j}\right)}_{x\bar{x}}\\ \; & +q\left(g\left(v_{j}^{n+\frac{1}{2}},w_{j}^{n+\frac{1}{2}}\right)-{\hat{\Theta }}_{j}\right)={\hat{R}}_{F,j}^{n}, \end{array}$$

(24) $$\begin{array}{cc} {\left(e_{F,j}\right)}_{t}^{n}+\frac{h^{2}}{12}{\left(e_{F,j}\right)}_{t,x\bar{x}}^{n} & +\eta {\left(E_{F,j}\right)}_{x\bar{x}}^{n+\frac{1}{2}}+\frac{qh^{2}}{12}{\left(g\left(w_{j}^{n+\frac{1}{2}},v_{j}^{n+\frac{1}{2}}\right)-{\tilde{\Theta }}_{j}\right)}_{x\bar{x}}\\ \; & +q\left(g\left(w_{j}^{n+\frac{1}{2}},v_{j}^{n+\frac{1}{2}}\right)-{\tilde{\Theta }}_{j}\right)={\tilde{R}}_{F,j}^{n}, \end{array}$$

where

$$\begin{array}{cc} g\left(v_{j}^{n+\frac{1}{2}},w_{j}^{n+\frac{1}{2}}\right)-{\hat{\Theta }}_{j}\; & ={\hat{g}}_{x}e_{F,j}^{n+\frac{1}{2}}+{\hat{g}}_{y}E_{F,j}^{n+\frac{1}{2}}+\frac{1}{2}{\hat{g}}_{xx}^{*}{\left(\frac{e_{C,j}^{n+1}+e_{F,j}^{n}}{2}\right)}^{2}\\ \; & +\frac{1}{2}{\hat{g}}_{yy}^{*}{\left(\frac{E_{C,j}^{n+1}+E_{F,j}^{n}}{2}\right)}^{2}+\frac{1}{2}{\hat{g}}_{xy}^{*}\left(\frac{e_{C,j}^{n+1}+e_{F,j}^{n}}{2}\frac{E_{C,j}^{n+1}+E_{F,j}^{n}}{2}\right),\\ g\left(w_{j}^{n+\frac{1}{2}},v_{j}^{n+\frac{1}{2}}\right)-{\tilde{\Theta }}_{j}\; & ={\tilde{g}}_{x}E_{F,j}^{n+\frac{1}{2}}+{\tilde{g}}_{y}e_{F,j}^{n+\frac{1}{2}}+\frac{1}{2}{\tilde{g}}_{xx}^{*}{\left(\frac{E_{C,j}^{n+1}+E_{F,j}^{n}}{2}\right)}^{2}\\ \; & +\frac{1}{2}{\tilde{g}}_{yy^{*}}{\left(\frac{e_{C,j}^{n+1}+e_{F,j}^{n}}{2}\right)}^{2}+\frac{1}{2}{\tilde{g}}_{xy}^{*}\left(\frac{e_{C,j}^{n+1}+e_{F,j}^{n}}{2}\frac{E_{C,j}^{n+1}+E_{F,j}^{n}}{2}\right), \end{array}$$

and

$$\begin{array}{cc} \; & {\bar{v}}_{j}^{n+\frac{1}{2}}=\frac{V_{C,j}^{n+1}+V_{F,j}^{n}}{2},{\bar{w}}_{j}^{n+\frac{1}{2}}=\frac{W_{C,j}^{n+1}+W_{F,j}^{n}}{2},\\ \; & {\hat{g}}_{x}=g_{x}\left({\bar{v}}_{j}^{n+\frac{1}{2}},{\bar{w}}_{j}^{n+\frac{1}{2}}\right),{\hat{g}}_{y}=g_{y}\left({\bar{v}}_{j}^{n+\frac{1}{2}},{\bar{w}}_{j}^{n+\frac{1}{2}}\right),\\ \; & {\hat{g}}_{xx}^{*}=g_{xx}\left(v_{j}^{*},w_{j}^{*}\right),{\hat{g}}_{yy}^{*}=g_{yy}\left(v_{j}^{*},w_{j}^{*}\right),{\hat{g}}_{xy}^{*}=g_{xy}\left(v_{j}^{*},w_{j}^{*}\right),\\ \; & {\tilde{g}}_{x}=g_{x}\left({\bar{w}}_{j}^{n+\frac{1}{2}},{\bar{v}}_{j}^{n+\frac{1}{2}}\right),{\tilde{g}}_{y}=g_{y}\left({\bar{w}}_{j}^{n+\frac{1}{2}},{\bar{v}}_{j}^{n+\frac{1}{2}}\right),\\ \; & {\tilde{g}}_{xx}^{*}=g_{xx}\left(w_{j}^{*},v_{j}^{*}\right),{\tilde{g}}_{yy}^{*}=g_{yy}\left(w_{j}^{*},v_{j}^{*}\right),{\tilde{g}}_{xy}^{*}=g_{xy}\left(w_{j}^{*},v_{j}^{*}\right). \end{array}$$

Based on the above set of error equations, we then obtain the following error estimation of the fine mesh solution.

**Theorem** **2.** *Suppose that the exact solution $u^{n}=v^{n}+iw^{n}$ to the initial boundary value problem Equation (1) is sufficiently smooth and let $U_{F}^{n}=V_{F}^{n}+iW_{F}^{n}$ be the numerical solution on the time fine mesh. Then, there exists a positive constant M independent of $h,\tau_{C},\tau_{F}$ such that*

$$∥u^{n}-U_{F}^{n}{∥}_{L_{2}}\leq M\left(\tau_{F}^{2}+\tau_{C}^{4}+h^{4}\right).$$

**Proof.**  Omitting the subindex *j* and taking the inner product (·,·) on both sides of Equation (23) with $E_{F}^{n+1}+E_{F}^{n}$, we have

(25) $$\begin{array}{cc} \; & \frac{1}{\tau_{F}}\left[∥E_{F}^{n+1}{∥}_{L_{2}}^{2}-{∥E_{F}^{n}∥}_{L_{2}}^{2}\right]-\frac{h^{2}}{12\tau_{F}}\left[∥|E_{F,x}^{n+1}{|∥}_{L_{2}}^{2}-∥|E_{F,x}^{n}{|∥}_{L_{2}}^{2}\right]-2\eta \left(e_{F,x\bar{x}}^{n+\frac{1}{2}},E_{F}^{n+\frac{1}{2}}\right)\\ \; & \;=\frac{qh^{2}}{6}\left({\left({\hat{g}}_{x}e_{F}^{n+\frac{1}{2}}\right)}_{x\bar{x}},E_{F}^{n+\frac{1}{2}}\right)+2q\left({\hat{g}}_{x}e_{F}^{n+\frac{1}{2}},E_{F}^{n+\frac{1}{2}}\right)\\ \; & \;+\frac{qh^{2}}{6}\left({\left({\hat{g}}_{y}E_{F}^{n+\frac{1}{2}}\right)}_{x\bar{x}},E_{F}^{n+\frac{1}{2}}\right)+2q\left({\hat{g}}_{y}E_{F}^{n+\frac{1}{2}},E_{F}^{n+\frac{1}{2}}\right)\\ \; & \;+\frac{qh^{2}}{12}\left({\left({\hat{g}}_{xx}^{*}{\left(\frac{e_{C}^{n+1}+e_{F}^{n}}{2}\right)}^{2}\right)}_{x\bar{x}},E_{F}^{n+\frac{1}{2}}\right)+q\left({\hat{g}}_{xx}^{*}{\left(\frac{e_{C}^{n+1}+e_{F}^{n}}{2}\right)}^{2},E_{F}^{n+\frac{1}{2}}\right)\\ \; & \;\;+\frac{qh^{2}}{12}\left({\left({\hat{g}}_{yy}^{*}{\left(\frac{E_{C}^{n+1}+E_{F}^{n}}{2}\right)}^{2}\right)}_{x\bar{x}},E_{F}^{n+\frac{1}{2}}\right)+q\left({\hat{g}}_{yy}^{*}{\left(\frac{E_{C}^{n+1}+E_{F}^{n}}{2}\right)}^{2},E_{F}^{n+\frac{1}{2}}\right)\\ \; & \;+\frac{qh^{2}}{6}\left({\left({\hat{g}}_{xy}^{*}\left(\frac{e_{C}^{n+1}+e_{F}^{n}}{2}\frac{E_{C}^{n+1}+E_{F}^{n}}{2}\right)\right)}_{x\bar{x}},E_{F}^{n+\frac{1}{2}}\right)+q\left({\hat{g}}_{xy}^{*}\left(\frac{e_{C}^{n+1}+e_{F}^{n}}{2}\frac{E_{C}^{n+1}+E_{F}^{n}}{2}\right),E_{F}^{n+\frac{1}{2}}\right)\\ \; & \;-2\left({\tilde{R}}_{F}^{n},E_{F}^{n+\frac{1}{2}}\right)=\sum_{i=1}^{10}T_{i}-2\left({\tilde{R}}_{F}^{n},E_{F}^{n+\frac{1}{2}}\right), \end{array}$$

For each term on the right side of Equation (25), we estimate them as follows:

(26) $$\begin{array}{ll} |T_{1}| & =|\frac{qh^{2}}{6}\left({\left({\hat{g}}_{x}e_{F}^{n+\frac{1}{2}}\right)}_{x\bar{x}},E_{F}^{n+\frac{1}{2}}\right)|\\ \; & =|-\frac{qh^{2}}{6}\left({\left({\hat{g}}_{x}e_{F}^{n+\frac{1}{2}}\right)}_{x},E_{F,x}^{n+\frac{1}{2}}\right)|\leq Mh^{2}∥|{\left({\hat{g}}_{x}e_{F}^{n+\frac{1}{2}}\right)}_{x}{|∥}_{L_{2}}∥|E_{F,x}^{n+\frac{1}{2}}{|∥}_{L_{2}}\\ \; & \leq M∥{\hat{g}}_{x}e_{F}^{n+\frac{1}{2}}{∥}_{L_{2}}∥E_{F}^{n+\frac{1}{2}}∥\leq M∥e_{F}^{n+\frac{1}{2}}{∥}_{L_{2}}{∥E_{F}^{n+\frac{1}{2}}∥}_{L_{2}}, \end{array}$$

(27) $$\begin{array}{cc} |T_{2}| & =|2q\left({\hat{g}}_{x}e_{F}^{n+\frac{1}{2}},E_{F}^{n+\frac{1}{2}}\right)|\leq M∥e_{F}^{n+\frac{1}{2}}{∥}_{L_{2}}{∥E_{F}^{n+\frac{1}{2}}∥}_{L_{2}}, \end{array}$$

where the assumption $\max_{\left(x,y\right)\in R^{2}}|g_{x}\left|+\right|g_{y}|\leq L$, Cauchy–Schwarz inequality and Lemma 1 are used. Similarly, we also have

(28) $$\begin{array}{cc} \; & |T_{3}\left|+\right|T_{4}|\leq M∥E_{F}^{n+\frac{1}{2}}{∥}_{L_{2}}^{2},\\ \; & \sum_{i=5}^{10}|T_{i}|\leq M\left\{∥{\left(e_{C}^{n+1}\right)}^{2}{∥}_{L_{2}}+∥{\left(e_{F}^{n}\right)}^{2}{∥}_{L_{2}}+∥{\left(E_{C}^{n+1}\right)}^{2}{∥}_{L_{2}}+{∥{\left(E_{F}^{n}\right)}^{2}∥}_{L_{2}}\right\}{∥E_{F}^{n+\frac{1}{2}}∥}_{L_{2}},\\ \; & |2\left({\hat{R}}_{F}^{n},E_{F}^{n+\frac{1}{2}}\right)\left|\leq M\right|{\hat{R}}_{F}^{n}|∥E_{F}^{n+\frac{1}{2}}{∥}_{L_{2}}. \end{array}$$

Then, from Equations (26)–(28), we obtain

(29) $$\begin{array}{cc} {}[∥E_{F}^{n+1}{∥}_{L_{2}}^{2} & -∥E_{F}^{n}{∥}_{L_{2}}^{2}]-\frac{h^{2}}{12}\left[∥|E_{F,x}^{n+1}{|∥}_{L_{2}}^{2}-∥|E_{F,x}^{n}{|∥}_{L_{2}}^{2}\right]-2\tau_{F}\eta \left(e_{F,x\bar{x}}^{n+\frac{1}{2}},E_{F}^{n+\frac{1}{2}}\right)\\ \; & \leq M\tau_{F}\{∥E_{F}^{n+\frac{1}{2}}{∥}_{L_{2}}^{2}+{∥e_{F}^{n+\frac{1}{2}}∥}_{L_{2}}^{2}\\ \; & +∥{\left(e_{C}^{n+1}\right)}^{2}{∥}_{L_{2}}^{2}+∥{\left(e_{F}^{n}\right)}^{2}{∥}_{L_{2}}^{2}+∥{\left(E_{C}^{n+1}\right)}^{2}{∥}_{L_{2}}^{2}+∥{\left(E_{F}^{n}\right)}^{2}{∥}_{L_{2}}^{2}+{\left|{\hat{R}}_{F}^{n}\right|}^{2}\}. \end{array}$$

Taking the inner product on both sides of Equation (24) with $e_{F}^{n+1}+e_{F}^{n}$, and then in an entirely analogous manner, a similar estimate may be obtained as follows.

(30) $$\begin{array}{cc} {}[∥e_{F}^{n+1}{∥}_{L_{2}}^{2} & -∥e_{F}^{n}{∥}_{L_{2}}^{2}]-\frac{h^{2}}{12}\left[∥|e_{F,x}^{n+1}{|∥}_{L_{2}}^{2}-∥|e_{F,x}^{n}{|∥}_{L_{2}}^{2}\right]+2\tau_{F}\eta \left(E_{F,x\bar{x}}^{n+\frac{1}{2}},e_{F}^{n+\frac{1}{2}}\right)\\ \; & \leq M\tau_{F}\{∥E_{F}^{n+\frac{1}{2}}{∥}_{L_{2}}^{2}+{∥e_{F}^{n+\frac{1}{2}}∥}_{L_{2}}^{2}\\ \; & +∥{\left(e_{C}^{n+1}\right)}^{2}{∥}_{L_{2}}^{2}+∥{\left(e_{F}^{n}\right)}^{2}{∥}_{L_{2}}^{2}+∥{\left(E_{C}^{n+1}\right)}^{2}{∥}_{L_{2}}^{2}+∥{\left(E_{F}^{n}\right)}^{2}{∥}_{L_{2}}^{2}+{\left|{\tilde{R}}_{F}^{n}\right|}^{2}\}. \end{array}$$

Adding Equations (29) and (30) and summing from n=0 to *m*, we obtain

$$\begin{array}{cc} ∥u^{m}-U_{F}^{m}{∥}_{L_{2}}^{2} & \leq M\tau_{F}\sum_{p=0}^{m-1}{∥u^{p}-U_{F}^{p}∥}_{L_{2}}^{2}+M\tau_{F}\sum_{p=0}^{m}\left(∥{\left(e_{C}^{p}\right)}^{2}{∥}_{L_{2}}^{2}+{∥{\left(E_{C}^{p}\right)}^{2}∥}_{L_{2}}^{2}\right)\\ \; & +M\tau_{F}\sum_{p=0}^{m-1}{\left|R_{F}^{p}\right|}^{2}+M\tau_{F}\sum_{p=0}^{m-1}\left(∥{\left(e_{F}^{p}\right)}^{2}{∥}_{L_{2}}^{2}+{∥{\left(E_{F}^{p}\right)}^{2}∥}_{L_{2}}^{2}\right). \end{array}$$

Here we have used Lemma 1 and the assumption that $\tau_{F}$ is small enough. Furthermore, we use the technique shown in [48,49] and Lemma 2 to obtain the conclusion. □

## 5. Numerical Results

In this section, we present two numerical examples to illustrate the efficiency of the algorithm discussed in Section 4.

### 5.1. Single Soliton Solution to the NLS Equation

In order to test the accuracy of the algorithm presented in Section 4, Equation (1) with the coefficient η=1,q=2 and the exact solution [24]

u(x,t)=sech(x−4t)exp(2ix−3it)

is solved by time two-mesh (TT-M) CD scheme Equations (6)–(12) and standard nonlinear implicit (SNI) CD scheme Equations (6)–(8) at the domain of (t,x)∈(0,1]×[−30,30], respectively. The NLS Equation (1) has an infinite number of conservation laws including, e.g.,

$$\begin{array}{cc} \; & Q=\int_{-\infty }^{+\infty }{\left|u\left(x,t\right)\right|}^{2}dx=\int_{-\infty }^{+\infty }{\left|\varphi \left(x\right)\right|}^{2}dx,\\ \; & E=\int_{-\infty }^{+\infty }\left(|\frac{\partial u}{\partial x}|-\frac{q}{2}{\left|u\right|}^{4}\right)dx=\int_{-\infty }^{+\infty }\left(|\frac{\partial \varphi }{\partial x}|-\frac{q}{2}{\left|\varphi \right|}^{4}\right)dx. \end{array}$$

Further, the numerical invariants are evaluated as

$$Q≃\sum_{j=0}^{J-1}{\left|U_{j}^{n}\right|}^{2}h,\;\;E≃\sum_{j=0}^{J-1}\left[|{\left(U_{j}\right)}_{x}^{n}{|}^{2}-\frac{q}{2}{\left|U_{j}^{n}\right|}^{4}\right]h.$$

Let $U_{F}$ and $U_{S}$ be the numerical solutions of the TT-MCD scheme and the SNI-CD scheme, respectively. Here, we also used some notations

$$\begin{array}{cc} \; & E_{TT-M}\left(\tau ,h\right)=∥u^{N}-U_{F}^{N}{∥}_{L_{2}},\;\;E_{SNI}\left(\tau ,h\right)={∥u^{N}-U_{S}^{N}∥}_{L_{2}},\\ \; & Rate_{TT-M}^{t}=\log_{2}\left(\frac{E_{TT-M}\left(2\tau ,h\right)}{E_{TT-M}\left(\tau ,h\right)}\right),\;\;Rate_{TT-M}^{x}=\log_{2}\left(\frac{E_{TT-M}\left(\tau ,2h\right)}{E_{TT-M}\left(\tau ,h\right)}\right) \end{array}$$

to denote the convergence rates of the TT-MCD scheme in time and space, respectively. Similarly, notations $Rate_{SNI}^{t}$ and $Rate_{SNI}^{x}$ are defined in the SNI-CD scheme.

In Table 1, the discrete $L_{2}$-norm errors, convergence rates in time and the time cost of the TT-MCD scheme and the SNI-CD scheme are given, respectively. These data are obtained by taking fixed the space step h=0.02 and changed the time step $\tau_{C}=5\tau_{F}=1/4,$1/8,1/16,1/32. Further, Table 2 presents the discrete $L_{2}$-norm errors, convergence rates in space and the time cost of the two schemes by taking fixed $\tau_{C}=1/50,\;\tau_{F}=1/2500$ and changed h=1/2,1/4,1/8,1/16. From Table 1 and Table 2, we observe that the errors of the two schemes are almost identical and the temporal and spatial convergence rates of the both schemes are nearly approach two and four, respectively, which are in agreement with our theoretical results. Furthermore, from Table 1 and Table 2, one can also clearly that our TT-MCD scheme has much lower time cost than the SNI-CD scheme.

**Figure 1:**
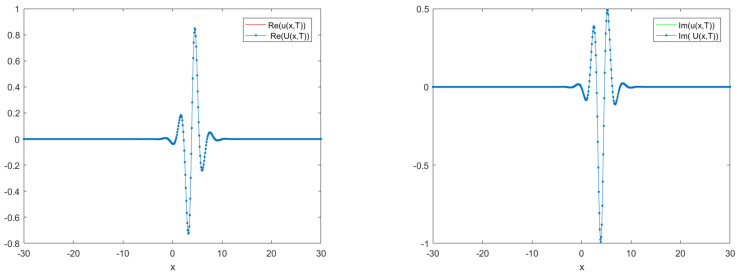
Real (**left**) and imaginary (**right**) parts of exact and numerical solutions at t=1 with $\tau_{C}=0.1,$$\tau_{F}=0.01,h=0.1$.

**Figure 2:**
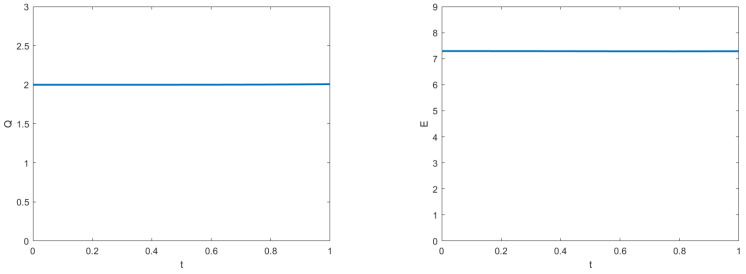
Numerical charge (Q) and energy (E) from t=0 to 1 with $\tau_{C}=0.1,\tau_{F}=0.01,h=0.1$.

**Figure 3:**
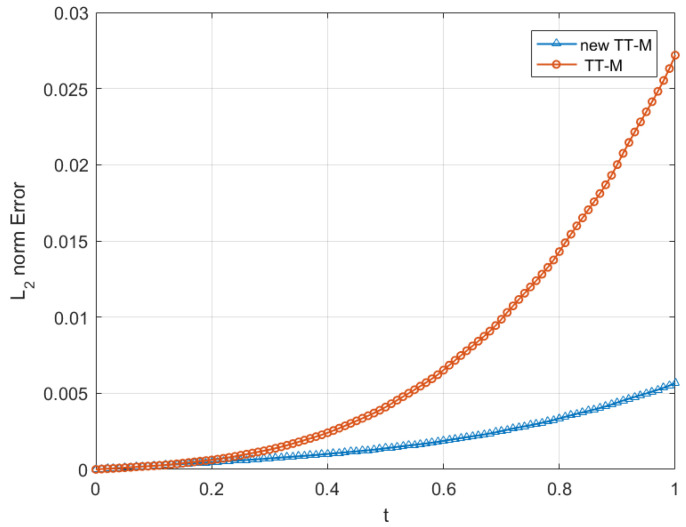
Comparison of $L_{2}$ norm errors of the original TT-M method and its of the proposed TT-M method at t=1.

The curves of real and imaginary parts of the TT-MCD and exact solutions at t=1.0 are shown in Figure 1, which indicates that our numerical solution and the exact solution are in good agreement. The numerical invariant values are plotted in Figure 2, which shows that our method preserves the two conservation laws. A comparison of $L_{2}$ norm errors of the original TT-M method and its of the proposed TT-M method at t=1 with $\tau_{C}=0.1,\tau_{F}=0.01,h=0.1$ is shown in Figure 3, which implies that our method can improve the calculation accuracy of fine grid solutions.

### 5.2. The Interaction of Two Solitons for the NLS Equation

In this test problem, we investigate the interaction of two solitary wave propagation. The initial and boundary conditions are, respectively, given by [17]

u(x,0)=sech(x+15)exp(2ix)+1.5sech(1.5x−7.5)exp(−2ix),

and

u(−30,t)=u(30,t)=0.

**Figure 4:**
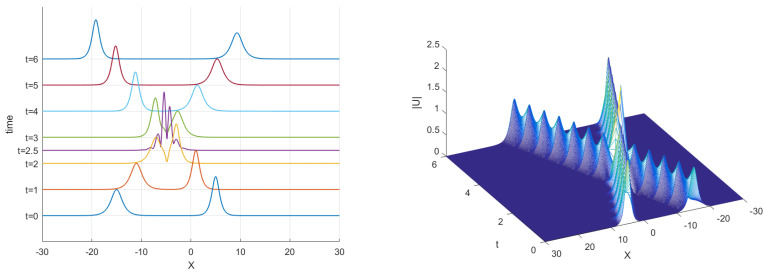
2D (**left**) and 3D (**right**) graphics of collision of double solitons with $\tau_{C}=0.03,$$\tau_{F}=0.01,h=0.1$.

**Figure 5:**
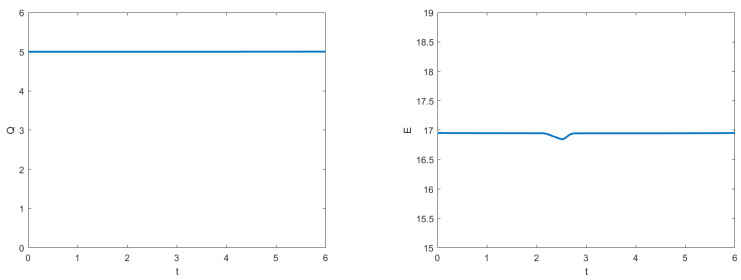
Numerical charge (Q) and energy (E) from t=0 to 6 with $\tau_{C}=0.03,\tau_{F}=0.01,h=0.1$.

This initial condition yields a two-soliton solution. As a result of solitons, after the collision, double solitons preserve their properties such as shape, velocity, and amplitudes, which will be observed at the simulations of double solitons are given in Figure 4. The numerical experiment is performed from t=0 to t=6 with $\tau_{C}=0.03,\tau_{F}=0.01,h=0.1$. As time progresses, the two solitons are traveling in opposite directions. The faster soliton eventually catches up with the slower one. Then, the two solitons collide and separate, but recover their shapes afterward despite a strongly nonlinear interaction. These results are in qualitative agreement with the behavior predicted by the soliton theory [6]. The evolution of the two invariants is given in Figure 5, in which the charge *Q* seems to remain constant all the time. The energy *E* also keeps constant except for a slight change that occurs during the interaction of two solitons. The results given in Figure 5 show that our method preserves the conservation laws for charge and energy.

### 5.3. Birth of Standing Soliton with the Maxwellian Initial Condition

Theory predict that if

$$\int_{\infty }^{\infty }u\left(x,0\right)dx\geq \pi ,$$

a soliton u(x,t) will appear over time, otherwise the soliton decay away [24]. Here, we consider the birth of soliton with the Maxwellian initial condition [24]:

$$u\left(x,0\right)=A\exp\left(-x^{2}\right),\;-45\leq x\leq 45.$$

**Figure 6:**
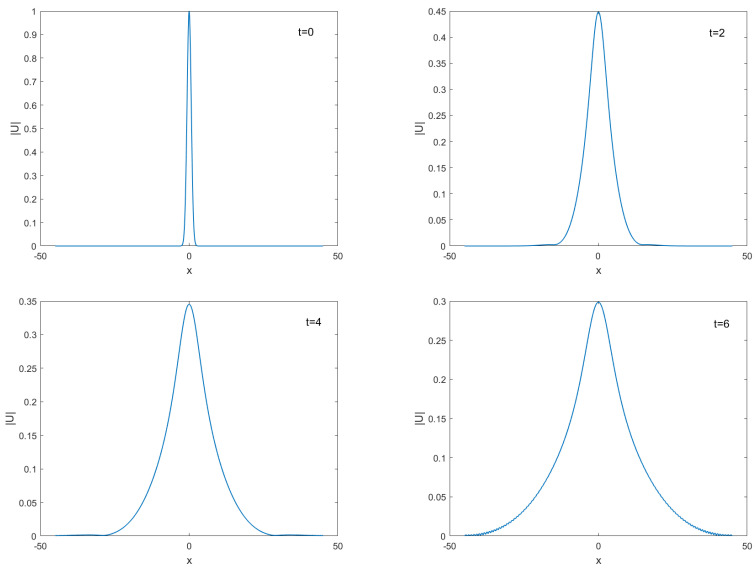
Birth of standing solitons for A=1 at different time t=0,2,4, and t=6.

**Figure 7:**
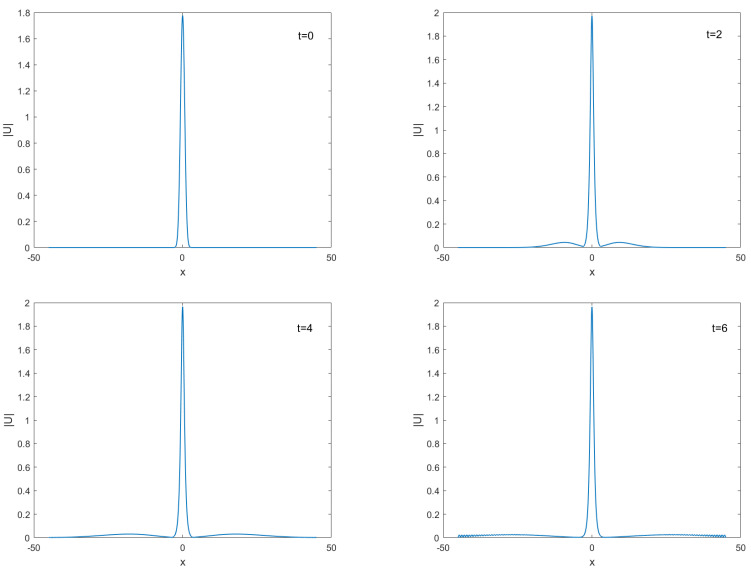
Birth of standing solitons for A=1.78 at different time t=0,2,4, and t=6.

**Figure 8:**
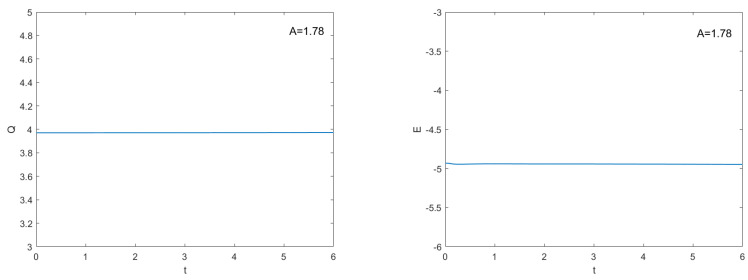
Numerical charge (Q) and energy (E) from t=0 to 6 for A=1.78.

The values of all parameters are chosen to be $h=0.08,\tau_{C}=0.025,\tau_{F}=0.005$ and q=2 to exhibit the behaviors of solutions for A=1 and A=1.78 and time running up from t=0 to t=6 are given in Figure 6 and Figure 7, respectively. It is seen from Figure 6 and Figure 7 that the approximate solution of |u| decay by time increases for A=1 unless for A=1.78 soliton’s amplitude, shape, and speed are protected, and the locations of solitons do not change for both cases. In Figure 8, the numerical invariants *Q* and *E* for A=1.78 are plotted. As it is seen undoubtedly from Figure 8 that TT-MCD method produces charge *Q* and energy *E* are almost constant.

## 6. Conclusions

In this article, a TT-MCD algorithm is presented for the one-dimensional nonlinear Shördinger equation. This new TT-MCD algorithm contains three steps of a nonlinear implicit CD system on the time coarse mesh of size $\tau_{C}$, some useful values on the time fine mesh of size $\tau_{F}$, and a linear system on the time fine mesh. Especially, the fine mesh solution, which becomes available, is also used as the initial guess in the third step to improve the calculation accuracy of fine grid solutions. The discrete $L_{2}$-norm convergence result with $O(\tau_{C}^{4}+\tau_{F}^{2}+h^{4})$ is proved in detail. Three different test problems have been investigated. The performance and accuracy of the algorithm have been shown by investigating calculating the error discrete $L_{2}$-norm and two conservation laws of charge and energy and their relative changes. The obtained results show that the new TT-MCD algorithm can produce numerical solutions of the NLS equation with high accuracy and preserve the conservation laws of charge and energy. Moreover, the computing cost of the TT-MCD method is less than that of the standard nonlinear implicit (SNI) CD scheme. In the near future, we will focus on the extension of the time two-mesh idea to multidimensional nonlinear Shördinger equations combining with alternating direction implicit (ADI) or locally one-dimensional (LOD) techniques.

## Figures and Tables

**Table 1:** The errors and convergence rates in time with $h=0.02,\tau_{C}=5\tau_{F}$.

$\left(\tau_{C},\tau_{F}\right)$	$E_{\mathrm{TT}-M}\left(\tau ,h\right)$	${\mathrm{Rate}}_{\mathrm{TT}-M}^{t}$	CPU Time (s)	$E_{\mathrm{SNI}}\left(\tau_{F},h\right)$	${\mathrm{Rate}}_{\mathrm{SNI}}^{t}$	CPU Time (s)
$\left(\frac{1}{4},\frac{1}{20}\right)$	$7.2763\times 10^{-2}$	–	139.9	$5.5807\times 10^{-2}$	–	332.6
$\left(\frac{1}{8},\frac{1}{40}\right)$	$1.5492\times 10^{-2}$	2.2317	278.6	$1.3867\times 10^{-2}$	2.0088	678.4
$\left(\frac{1}{16},\frac{1}{80}\right)$	$3.1757\times 10^{-3}$	2.2864	568.6	$3.4604\times 10^{-3}$	2.0027	1253.0
$\left(\frac{1}{32},\frac{1}{160}\right)$	$8.0260\times 10^{-4}$	1.9843	1138.6	$8.6496\times 10^{-4}$	2.0002	2195.9

**Table 2:** The errors and convergence rates in space with $\tau_{C}=1/50$ and $\tau_{F}=1/2500$.

*h*	$E_{\mathrm{TT}-M}\left(\tau ,h\right)$	${\mathrm{Rate}}_{\mathrm{TT}-M}^{x}$	CPU Time (s)	$E_{\mathrm{SNI}}\left(\tau_{F},h\right)$	${\mathrm{Rate}}_{\mathrm{SNI}}^{x}$	CPU Time (s)
$\frac{1}{2}$	$2.3706\times 10^{-1}$	–	28.8	$2.3718\times 10^{-1}$	–	51.6
$\frac{1}{4}$	$1.1128\times 10^{-2}$	4.4130	85.8	$1.1136\times 10^{-2}$	4.4127	199.9
$\frac{1}{8}$	$6.7349\times 10^{-4}$	4.0464	291.3	$6.7723\times 10^{-4}$	4.0395	924.1
$\frac{1}{16}$	$4.2889\times 10^{-5}$	3.9730	1023.9	$4.5251\times 10^{-5}$	3.9036	3751.5

## Data Availability

All the data were computed using our algorithm.

## References

[1] Bao W., Cai Y. (2013). Mathematical theory and numerical methods for Bose-Einstein condensation, Kinet. Relat. Model..

[2] Akhmediev N., Soto-Crespo J.M., Ankiewicz A. (2009). How to excite a rogue wave. Phys. Rev. A.

[3] Akhmediev N., Eleonskii V.M., Kulagin N.E. (1987). Exact first-order solutions of the nonlinear Schrödinger equation. Theor. Math. Phys..

[4] Nikolić S.N., Aleksixcx N.B., Ashour O.A., Belixcx M.R., Chin S.A. (2017). Systematic generation of higher-order solitons and breathers of the Hirota equation on different backgrounds. Nonlinear Dyn..

[5] Ankiewicz A., Soto-Crespo J.M., Akhmediev N. (2010). Rogue waves and rational solutions of the Hirota equation. Phys. Rev. E.

[6] Ablowitz M.J., Prinari B., Trubatch A.D. (2005). Discrete and continuous nonlinear Schrödinger systems. Bull. New Ser. Am. Math. Soc..

[7] Xie S.S., Li G.X., Yi S. (2009). Compact finite difference schemes with high accuracy for one-dimensional nonlinear Schrödinger equation. Comput. Methods Appl. Mech Engrg..

[8] Akrivis G.D. (1993). Finite difference discretization of the cubic Schrödinger equation. IMA J. Numer. Anal..

[9] Dehghan M., Taleei A. (2010). A compact split-step finite difference method for solving the nonlinear Schrödinger equations with constant and variable coefficients. Comput. Phys. Commun..

[10] Bao W.Z., Cai Y.Y. (2012). Uniform error estimates of finite difference methods for the nonlinear Schrödinger equation with wave operator. SIAM J. Numer. Anal..

[11] Li X., Zhang L., Wang S. (2012). A compact finite difference scheme for the nonlinear Schrödinger equation with wave operator. Appl. Math. Comput..

[12] Wang T., Guo B., Xu Q. (2013). Fourth-order compact and energy conservative difference schemes for the nonlinear Schrödinger equation in two dimensions. J. Comput. Phys..

[13] Liao H., Sun Z., Shi H. (2010). Maximum norm error analysis of explicit schemes for two-dimensional nonlinear Schrödinger equations. Sci. Sin. Math..

[14] Wang H. (2005). Numerical studies on the split-step finite difference method for nonlinear Schrödinger equations. Appl. Math. Comput..

[15] Kong L.H., Duan Y.L., Wang L., Yin X.L., Ma Y.P. (2012). Spectral-like resolution compact ADI finite difference method for the multi-dimensional Schrödinger equations. Math. Comput. Model..

[16] Eskar R., Feng X.L., Huang P.Z. (2018). Fourth-order compact split-step finite difference method for solving the two and three-dimensional nonlinear Schrödinger equations. Adv. Appl. Math. Mech..

[17] Delfour M., Fortin M., Payer G. (1981). Finite difference solutions of a Non-linear Schrödinger equation. J. Comput. Phys..

[18] Karakashian O., Makridakis C. (1998). A space-time finite element method for the nonlinear Schrödinger equation: The discontinuous Galerkin method. Math. Comp..

[19] Shi D.Y., Wang J.J. (2017). Unconditional Superconvergence Analysis of a Crank-Nicolson Galerkin FEM for Nonlinear Schrödinger Equation. J. Sci. Comput..

[20] Shi D.Y., Liao X., Wang L.L. (2017). A nonconforming quadrilateral finite element approximation to nonlinear Schrödinger equation. Acta Math. Sci..

[21] Wang J.L. (2014). A new error analysis of Crank-Nicolson Galerkin FEMs for a generalized nonlinear Schrödinger equation. J. Sci. Comput..

[22] Gong X., Shen L., Zhou A. (2008). Finite element approximations for Schrödinger equations with applications to electronic structure computations. J. Comput. Math..

[23] Bao W., Cai Y.Y. (2014). Uniform and optimal error estimates of an exponential wave integrator sine pseudospectral method for the nonlinear Schrödinger equation with wave operator. SIAM J. Numer. Anal..

[24] Xu Y., Shu C.W. (2005). Local discontinuous Galerkin methods for nonlinear Schrödinger equations. J. Comput. Phys..

[25] Li M., Zhao J.K., Wang N., Chen S.C. (2021). Conforming and nonconforming conservative virtual element methods for nonlinear Schrödinger equation: A unified framework. Comput. Methods Appl. Mech. Eng..

[26] Grinstein F.F., Rabitz H., Askar A. (1983). The multigrid method for accelerated solution of the discretized Schrödinger equation. J. Comp. Phys..

[27] Chang Q., Wang G. (1990). Multigrid and adaptive algorithm for solving the nonlinear Schrödinger equation. J. Comp. Phys..

[28] Ignat L., Zuazua E. (2009). Numerical dispersive schemes for the nonlinear Schrödinger equation. SIAM J. Numer. Anal..

[29] Jin J.C., Wei N., Zhang H.M. (2015). A two-grid finite-element method for the nonlinear Schrödinger equation. J. Comput. Math..

[30] Chien C.S., Huang H.T., Jeng B.W., Li Z.C. (2008). Two-grid discretization schemes for nonlinear Schrödinger equations. J. Comput. Appl. Math..

[31] Wu L. (2012). Two-grid mixed finite-element methods for nonlinear Schrödinger equations. Numer. Meth. Part. Differ. Equ..

[32] Hu H.Z., Chen Y.P. (2020). Numerical solution of two-dimensional nonlinear Schrödinger equation using a new two-grid finite element method. J. Comput. Appl. Math..

[33] Tian Z.K., Chen Y.P., Huang Y.Q., Wang J.Y. (2019). Two-grid method for the two-dimensional time-dependent Schrödinger equation by the finite element method. Comput. Math. Appl..

[34] Zhang H.M., Yin J.H., Jin J.C. (2021). A two-grid finite-volume method for the Schrödinger equation. Adv. Appl. Math. Mech..

[35] Chen C.J., Lou Y.Z., Hu H.Z. (2022). Two-grid finite volume element method for the time-dependent Schrödinger equation. Comput. Math. Appl..

[36] Wang J.J., Li M., Guo L.J. (2021). Superconvergence analysis for nonlinear Schrödinger equation with two-grid finite element method. Appl. Math. Lett..

[37] Ignat L., Zuazua E. (2005). A two-grid approximation scheme for nonlinear Schrödinger equations: Dispersive properties and convergence. C. R. Acad. Sci. Paris Ser. I.

[38] Xu J. (1994). A novel two-grid method for semilinear elliptic equations. SIAM J. Sci. Comput..

[39] Dawson C.N., Wheeler M.F., Woodward C.S. (1998). A two-grid finite difference scheme for nonlinear parabolic equations. SIAM J. Numer. Anal..

[40] Rui H., Liu W. (2015). A two-grid block-centered finite difference method for Darcy-Forchheimer flow in porous media. SIAM J. Numer. Anal..

[41] Li X., Rui H. (2017). A two-grid block-centered finite difference method for the nonlinear time-fractional parabolic equation. J. Sci. Comput..

[42] Liu Y., Yu Z.D., Li H., Liu F.W., Wang J.F. (2018). Time two-mesh algorithm combined with finite element method for time fractional water wave model. Int. J. Heat Mass Transf..

[43] Yin B.L., Liu Y., Li H., He S. (2019). Fast algorithm based on TT-M FE system for space fractional Allen-Cahn equations with smooth and non-smooth solutions. J. Comput. Phys..

[44] Liu Y., Fan E.Y., Yin B.L., Li H., Wang J.F. (2020). TT-M finite element algorithm for a two-dimensional space fractional Gray-Scott model. Comput. Math. Appl..

[45] Wen C., Liu Y., Yin B.L., Li H., Wang J.F. (2021). Fast second-order time two-mesh mixed finite element method for a nonlinear distributed-order sub-diffusion model. Numer. Algorithms.

[46] Qiu W.L., Xu D., Guo J., Zhou J. (2020). A time two-grid algorithm based on finite difference method for the two-dimensional nonlinear time-fractional mobile/immobile transport model. Numer. Algorithms.

[47] Xu D., Guo J., Qiu W.L. (2020). Time two-grid algorithm based on finite difference method for two-dimensional nonlinear fractional evolution equations. Appl. Numer. Math..

[48] Niu Y.X., Liu Y., Li H., Liu F.W. (2021). Fast high-order compact difference scheme for the nonlinear distributed-order fractional Sobolev model appearing in porous media.

[49] Chai L., Liu Y., Li H., Gao W. (2022). Fast TT-M fourth-order compact difference schemes for a two-dimensional space fractional Gray-Scott model.

